# Genomic surveillance of *Acinetobacter baumannii* in the Philippines, 2013–2014

**DOI:** 10.5365/wpsar.2021.12.4.863

**Published:** 2021-10-27

**Authors:** Jeremiah Chilam, Silvia Argimón, Marilyn T. Limas, Melissa L. Masim, June M. Gayeta, Marietta L. Lagrada, Agnettah M. Olorosa, Victoria Cohen, Lara T. Hernandez, Benjamin Jeffrey, Khalil Abudahab, Charmian M. Hufano, Sonia B. Sia, Matthew T.G. Holden, John Stelling, David M. Aanensen, Celia C. Carlos, on behalf of the Philippines Antimicrobial Resistance Surveillance Program

**Affiliations:** a Antimicrobial Resistance Surveillance Reference Laboratory, Research Institute for Tropical Medicine, Department of Health, Muntinlupa, Philippines.; b Centre for Genomic Pathogen Surveillance, Wellcome Genome Campus, Hinxton, England.; c University of St Andrews School of Medicine, St Andrews, Scotland.; d Brigham and Women’s Hospital, Boston, MA, USA.; e Big Data Institute, University of Oxford, Oxford, England.; † These authors contributed equally to this work.; * These authors contributed equally to this work.

## Abstract

**Objective:**

*Acinetobacter baumannii* is an opportunistic nosocomial pathogen that has increasingly become resistant to carbapenems worldwide. In the Philippines, rates of carbapenem resistance and multidrug resistance are above 50%. We undertook a genomic study of carbapenem-resistant *A. baumannii* in the Philippines to characterize the population diversity and antimicrobial resistance mechanisms.

**Methods:**

We sequenced the whole genomes of 117 *A. baumannii* isolates recovered by 16 hospitals in the Philippines between 2013 and 2014. From the genome sequences, we determined the multilocus sequence type, presence of acquired determinants of antimicrobial resistance and relatedness between isolates. We also compared the phenotypic and genotypic resistance results.

**Results:**

Carbapenem resistance was mainly explained by acquisition of the class-D β-lactamase gene bla^OXA-23^. The concordance between phenotypic and genotypic resistance to imipenem was 98.15%, and it was 94.97% overall for the seven antibiotics analysed. Twenty-two different sequence types were identified, including 7 novel types. The population was dominated by the high-risk international clone 2 (i.e. clonal complex 92), in particular by ST195 and ST208 and their single locus variants. Using whole-genome sequencing, we identified local clusters representing potentially undetected nosocomial outbreaks, as well as multihospital clusters that indicated interhospital dissemination. Comparison with global genomes suggested that the establishment of carbapenem-resistant international clone 2 in the Philippines is likely the result of clonal expansion and geographical dissemination, and at least partly explained by inadequate hospital infection control and prevention.

**Discussion:**

This is the first extensive genomic study of carbapenem-resistant *A. baumannii* in the Philippines, and it underscores the importance of hospital infection control and prevention measures to contain high-risk clones.

Hospital-acquired *Acinetobacter baumannii* infections are some of the most challenging to treat due to the bacterium’s ability to acquire resistance to different groups of antimicrobials and to survive for long periods on dry surfaces, making eradication in health care facilities difficult once it has become endemic. (*1*) A previous surveillance study in the Asia–Pacific area showed that Acinetobacter spp. was the organism most frequently isolated in ventilator-associated pneumonia, (*2*) while in recent years the Philippines Antimicrobial Resistance Surveillance Program (ARSP) has consistently reported *A. baumannii* as the second and third most commonly isolated organism from, respectively, cerebrospinal fluid and respiratory specimens. (*3*)

During the past two decades, *A. baumannii* has become increasingly resistant to carbapenems worldwide, with resistance rates of > 40% reported across several countries in the Asia–Pacific area, which is the highest prevalence of carbapenem resistance among important nosocomial Gram-negative pathogens. (*4*, *5*) This pattern is also observed in the Philippines, where the annual resistance rates for several antibiotics, including carbapenems, have been increasing, in 2017 reaching 56% for  meropenem and 57% for imipenem (**Fig. 1A–C**). In addition, the ARSP has reported rates of multidrug resistance of 63% for all isolates and 47% for blood isolates, with combined resistance to aminoglycosides, fluoroquinolones,  carbapenems and ampicillin-sulbactam. (*3*) Importantly, bacteraemia due to multidrug-resistant (MDR) *A. baumannii* has been shown to result in additional hospitalization and costs compared with bacteraemia due to non-MDR *A. baumannii*. (*6*)

**Figure 1:**
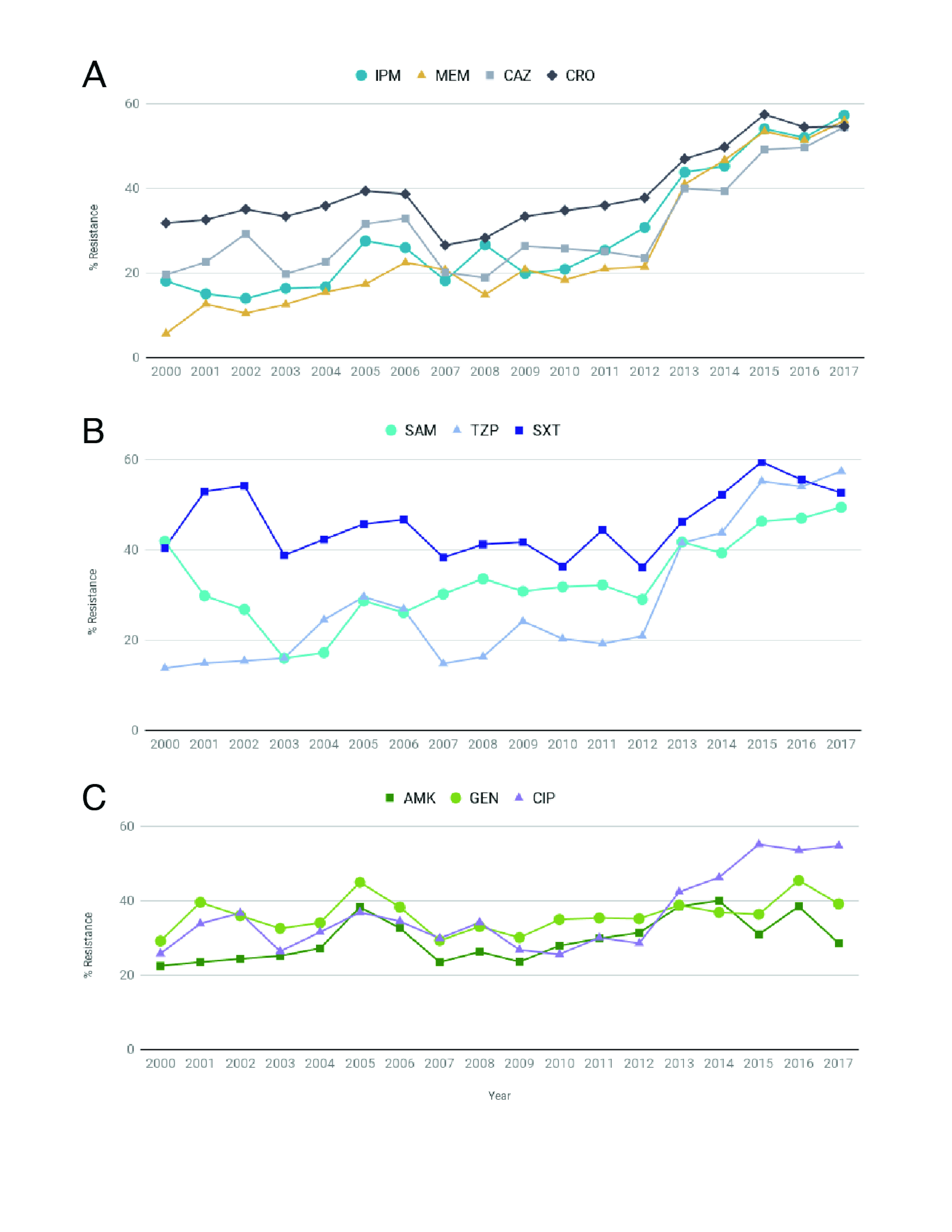
Annual resistance rates of Acinetobacter baumannii to different antibiotics, 2000–2017

[**insert** Figure 1]

Molecular typing methods have shown that clinical isolates of *A. baumannii* with an MDR phenotype belong mostly to two globally disseminated lineages: global clone (GC) 1 and GC2, also known as international clones (ICs) 1 and 2. Clonal complex 92 (CC92), corresponding to GC2, was the most prevalent in a previous study in nine Asian countries that included two isolates from the Philippines. (*7*)

The ARSP has been conducting surveillance of drug-resistant *A. baumannii* using phenotypic detection methods for bacterial identification and antimicrobial susceptibility testing. Whole-genome sequencing (WGS) can provide information on antimicrobial resistance (AMR) and genotyping with a single assay and with additional resolution to aid outbreak investigations. (*8*) Understanding the molecular epidemiology and AMR mechanisms of *A. baumannii* by monitoring the presence of international clones and the emergence of novel lineages in the Philippines can aid in the control of AMR. This report provides baseline data on the molecular epidemiology of *A. baumannii* in the Philippines, with a focus on the predominant circulating lineages and AMR mechanisms.

## Methods

### Bacterial isolates

A total of 5254 *A. baumannii* isolates were collected and tested for antimicrobial susceptibility by the ARSP’s sentinel sites from January 2013 to December 2014. Isolates resistant to carbapenems were subsequently referred to the Antimicrobial Resistance Surveillance Reference Laboratory for confirmation. Out of the 445 carbapenem-resistant *A. baumannii* isolates referred (155 in 2013 and 290 in 2014), 117 from 16 sentinel sites were selected for WGS according to the following criteria (previously described in detail): (*9*) (i) isolate was referred to the Reference Laboratory during 2013–2014; (ii) complete antimicrobial susceptibility data were available (i.e. a resistance profile); (iii) the overall prevalence of the resistance profile was in the ARSP database (including both referred and non-referred isolates); (iv) geographical representation of the different sentinel sites was present; (v) invasive isolates (i.e. from blood or cerebrospinal, joint, pleural or pericardial fluids) were selected when both invasive and non-invasive isolates were available for a combination of resistance profile, sentinel site and year of collection (**Table 1**). We used a proxy definition for “infection origin” whereby patients’ isolates collected on either of the first 2 days of hospitalization were categorized as from community-acquired infections, while isolates collected on hospital day 3 or later were categorized as from hospital-acquired infections.

**Table 1:** Total number of *A. baumannii* isolates analysed by the Antimicrobial Resistance Surveillance Program (ARSP) and referred to the Antimicrobial Resistance Surveillance Reference Laboratory (ARSRL) during 2013 and 2014, isolates submitted for whole-genome sequencing, and high-quality *A. baumannii* genomes obtained, discriminated by sentinel site and AMR profile

-	Number of isolates		
	2013	2014	Total
***A. baumannii* total ARSP**	2327	2927	5254
***A. baumannii* referred to ARSRL**	155	290	445
***A. baumannii* submitted for WGS**	59	58	117
***A. baumannii* high-quality genomes**	58	50	108
By sentinel site^a^	-	-	-
BGH	4	6	10
CMC	0	1	1
CVM	1	0	1
DMC	6	2	8
FEU	0	1	1
GMH	5	1	6
JLM	0	2	2
MAR	11	3	14
MMH	2	4	6
NKI	1	1	2
NMC	1	1	2
RMC	1	0	1
SLH	0	2	2
STU	3	3	6
VSM	13	19	32
ZMC	10	4	14
By AMR profile^b^	-	-	-
CAZ CRO IPM SAM TZP GEN AMK CIP SXT	48	36	84
CAZ CRO IPM SAM TZP GEN AMK CIP	0	6	6
CRO IPM SAM TZP AMK	3	1	4
CAZ CRO IPM SAM TZP GEN CIP	0	3	3
Susceptible	1	1	2
CAZ CRO SAM TZP GEN CIP SXT	1	0	1
IPM	0	1	1
CAZ CRO IPM SAM TZP AMK CIP SXT	1	0	1
CRO IPM TZP AMK	1	0	1
CAZ CRO SAM TZP GEN AMK	1	0	1
IPM TZP	0	1	1
CAZ CRO IPM SAM TZP GEN CIP SXT	1	0	1
CAZ CRO IPM SAM TZP	1	0	1
CAZ CRO IPM TZP	0	1	1

### Antimicrobial susceptibility testing

All *A. baumannii* isolates included in this study were tested for antimicrobial susceptibility to nine antibiotics representing six different classes: ceftazidime (CAZ), ceftriaxone (CRO), imipenem (IPM), ampicillin-sulbactam (SAM), piperacillin-tazobactam (TZP), gentamicin (GEN), amikacin (AMK), ciprofloxacin (CIP) and sulfamethoxazole-trimethoprim (SXT) (**Table 1**). Antimicrobial susceptibility was determined at the Reference Laboratory using one or a combination of the following methods: Kirby–Bauer disk diffusion; a gradient method, such as the E-Test (bioMérieux; Marcy-l’Étoile, France); or the Vitek 2 Compact automated system (bioMérieux; Marcy-l’Étoile, France). The zone of inhibition and minimum inhibitory concentration obtained were interpreted according to the twenty-sixth edition of the Clinical and Laboratory Standards Institute guidelines (*10*) to determine the resistance profile of the isolates as a list of antimicrobials to which the organism was not susceptible. MDR phenotypes were defined as nonsusceptibility to (*3*)1 agent in ^3^3 antimicrobial categories, and extensively drug-resistant (XDR) phenotypes were defined as nonsusceptibility to (*3*)1 agent in all but (*3*)2 classes.

### DNA extraction and whole-genome sequencing

DNA was extracted from a single colony of each of the 117 *A. baumannii* isolates using the QIAamp 96 DNA QIAcube HT Kit and the QIAcube HT system (Qiagen; Hilden, Germany). DNA extracts were multiplexed and sequenced on the Illumina HiSeq platform (Illumina; San Diego, CA, USA) with 100–base pair paired-end reads. Raw sequence data were deposited in the European Nucleotide Archive under the study accession PRJEB17615. Run accessions are provided through the links to Microreact projects in the figure legends.

### Bioinformatics analysis

Genome quality was assessed based on metrics produced for assemblies, annotation files and the alignment of the reads to the reference genome *A. baumannii* strain ATCC 17978 (GenBank accession CP000521), as previously described. (*9*) Annotated assemblies were produced from short-read Illumina data as previously described. (*11*)

We derived in silico the multilocus sequence type of the isolates from WGS. The sequence types were determined from assemblies using Pathogenwatch (https://pathogen.watch/) or from sequence reads using ARIBA (*12*) and the *A. baumannii* database hosted at PubMLST.org. (*13*) The isolates were assigned to international clones based on their sequence types, as previously described. (*14*-*17*)

Evolutionary relationships between isolates were inferred from single-nucleotide polymorphisms (SNPs) by mapping the paired-end reads to the reference genomes of *A. baumannii* strain A1 (accession CP010781) or AC29 (ST195, CC92, accession CP007535), as described in detail previously. (*9*) Mobile genetic elements were masked in the alignment of pseudogenomes with a script available at https://github.com/sanger-pathogens/remove_blocks_from_aln. Alignments of SNP positions were inferred with SNP-sites v. 2.4.1 (https://github.com/sanger-pathogens/snp-sites). (*18*) For the phylogenies of CC92 genomes, recombination regions detected with Gubbins (*19*) in the alignment of pseudogenomes were also removed. Maximum likelihood phylogenetic trees were generated with RAxML v. 8.28, (*20*) based on the generalized time reversible model with the GAMMA method of correction for among-site rate variation and 100 bootstrap replications. Pairwise SNP differences between primary isolates belonging to the same or different hospitals were calculated from alignments of SNP positions with a script available at https://github.com/simonrharris/pairwise_difference_count.

To contextualize the Philippine genomes, global *A. baumannii* genomes with geolocation data and an isolation date mainly between 2007 and 2017, for which raw Illumina paired-end sequence data were available at the European Nucleotide Archive, were downloaded, assembled and underwent quality control as described above. Evolutionary relationships between global genomes and those from this study were inferred from an alignment of SNP positions obtained after mapping the reads to the complete genome of strain A1 and masking regions with mobile genetic elements, as described above. The tree of 977 genomes was obtained using an approximately maximum–likelihood phylogenetic method with FastTree. (*21*) The tree of 573 global CC92 genomes was inferred with RAxML from an alignment of SNP sites obtained after mapping the genomes to the complete genome of strain AC29 and removing mobile genetic elements and recombination regions, as described above.

Known AMR determinants were identified from raw sequence reads using ARIBA (*12*) and two different AMR databases, a curated database of acquired resistance genes (*22*) and the Comprehensive Antibiotic Resistance Database (CARD). (*23*) Point mutations were identified on gyrase and topoisomerase genes with CARD and ARIBA, and corroborated with a literature search. The presence of the insertion sequences IS*Aba1* (GenBank accession AY758396) and IS*Aba125* (GenBank accession AY751533) upstream of the *ampC* gene was examined with ISMapper v. 2.0.1 (24) using the reference genome of *A. baumannii* A1 (GenBank accession CP010781) and default parameters. Genomic predictions of resistance were derived from the presence of known AMR genes and mutations identified in the genome sequences. The genomic predictions of AMR (the test) were compared with the phenotypic results (the reference), and the concordance between the two methods was computed for each of 7 antibiotics (756 total comparisons). For comparison purposes, isolates with either a resistant or an intermediate phenotype were considered nonsusceptible. An isolate with the same outcome for both the test and the reference (i.e. both susceptible or both nonsusceptible) was counted as a concordant isolate. The concordance was the number of concordant isolates over the total number of isolates assessed (expressed as a percentage).

All project data, including inferred phylogenies, AMR predictions and metadata are available through the web application Microreact (http://microreact.org).

## Results

### Demographic and clinical characteristics of the isolates

Out of the 117 *A. baumannii* genomes sequenced, 7 were excluded based on their quality, and 2 were identified in silico as *Acinetobacter pittii* (**Table 1**). The demographic and clinical characteristics of the remaining 108 *A. baumannii* isolates are summarized in **Table 2**. The age of the patients ranged from < 1 year to 92 years old, with 31.48% of the isolates (*n* = 34) from patients aged (*3*)65 years. Altogether 62.03% of the isolates (*n* = 67) were from males. The majority of the isolates were from inpatients (99.07%; *n* = 107) and were classified as being from a hospital-acquired infection (76.85%; *n* = 83). Respiratory samples (tracheal aspirates and sputum) accounted for 55.56% of the specimens (*n* = 60).

**Table 2:** Demographic and clinical characteristics of 108 sequenced and confirmed *A. baumannii* isolates collected from 16 ARSP sites

Characteristic		No. Isolates
**Sex**		
-	Male	67
-	Female	41
Age (in years)		
-	< 1	6
-	1–4	11
-	5–14	3
-	15–24	6
-	25–34	7
-	35–44	9
-	45–54	12
-	55–64	20
-	65–80	26
-	^3^81	8
Patient Type		
In-patient		107
Out-patient		1
Specimen Origin		
Community-acquired		25
Hospital-acquired		83
Submitted As*		
Carbapenem-resistant		104
Non carbapenem-resistant		4
Specimen Type		
Aspirate		1
Blood**		21
Bone		1
Catheter		1
Catheter, central		1
Cerebrospinal fluid**		13
Sputum		10
Tracheal aspirate		50
Ulcer		1
Urine		4
Wound		5

### Concordance between phenotypic and genotypic antimicrobial resistance

The genotypic predictions of AMR were highly concordant with the phenotypic results (overall concordance, 94.97%; **Table 3**). The concordance for imipenem was 98.15%, and of the 104 resistant isolates, 97 isolates from 14 hospitals (93.26%) carried the class D β-lactamase gene *bla***^OXA-23^** alone or in combination with *bla***^OXA-235^** (*n* = 1). The remaining isolates carried *bla***^NDM-6^** (*n* = 3), *bla***^NDM-1^** (*n* = 2) or *bla***^OXA-72^** (*n* = 2). One isolate had no known acquired carbapenemase. Of the 104 isolates resistant to imipenem, 89 (85.58%) were classified as XDR and 13 (12.50%) as MDR; also noted were the presence of the *armA* gene encoding a 16S rRNA methyltransferase, conferring broad-spectrum resistance to aminoglycosides in 54 isolates, and the co-occurrence of mutations in *gyrA* and *parC*, conferring resistance to fluoroquinolones in 95 isolates (**Table 3**). The mobilized colistin resistance gene (*mcr*) was not detected.

The isolates that were nonsusceptible to the third-generation cephalosporins ceftazidime (*n* = 99) or ceftriaxone (*n* = 104), or both, carried either the insertion sequence IS*Aba1* upstream of the chromosomal *bla***^ampC^** gene (*n* = 67), two or three copies of the *bla***^ampC^** gene (*n* = 22), the extended-spectrum β-lactamase genes *bla***^PER-1^** (*n* = 4) and *bla***^CTX-M-15^** (*n* = 1) or the carbapenemase gene *bla***^NDM^** (*n* = 5). Most of the false negative calls for ceftazidime (*n* = 3) and ceftriaxone (*n* = 8) (**Table 3**), for which no resistance mechanism was detected, coincided with intermediate susceptibility (*n* = 2 and *n* = 5, respectively).

### Genotypic findings

#### In silico genotyping

Multilocus sequence type was predicted in silico from the WGS data of the 108 *A. baumannii* isolates. A total of 22 different sequence types were identified from this data set as per the Oxford scheme, (*19*) 7 of which were novel and are now identified as ST2197, 2199, 2220, 2317, 2318, 2319 and 2320. The population was dominated by CC92 (*n* = 61), represented mainly by ST195 (*n* = 29) and ST208 (*n* = 23). CC92 was found at 13 of the 16 sentinel sites, with ST195 and ST208 spread geographically across 8 and 7 sentinel sites, respectively. In contrast, ST369 (*n* = 5) was found in only one site. The *armA* gene was found only in isolates belonging to CC92 (*n* = 54) and from 11 hospitals. Seven of the eight hospitals represented by six or more sequenced isolates showed clonal diversity, with at least two different circulating sequence types (**Table 4**), albeit with similar or identical resistance profiles. In contrast, all isolates collected by the Baguio General Hospital and Medical Center (BGH) belonged to sequence type 208.

#### *Population structure of* A. baumannii *in the Philippines*

**Figure 2:**
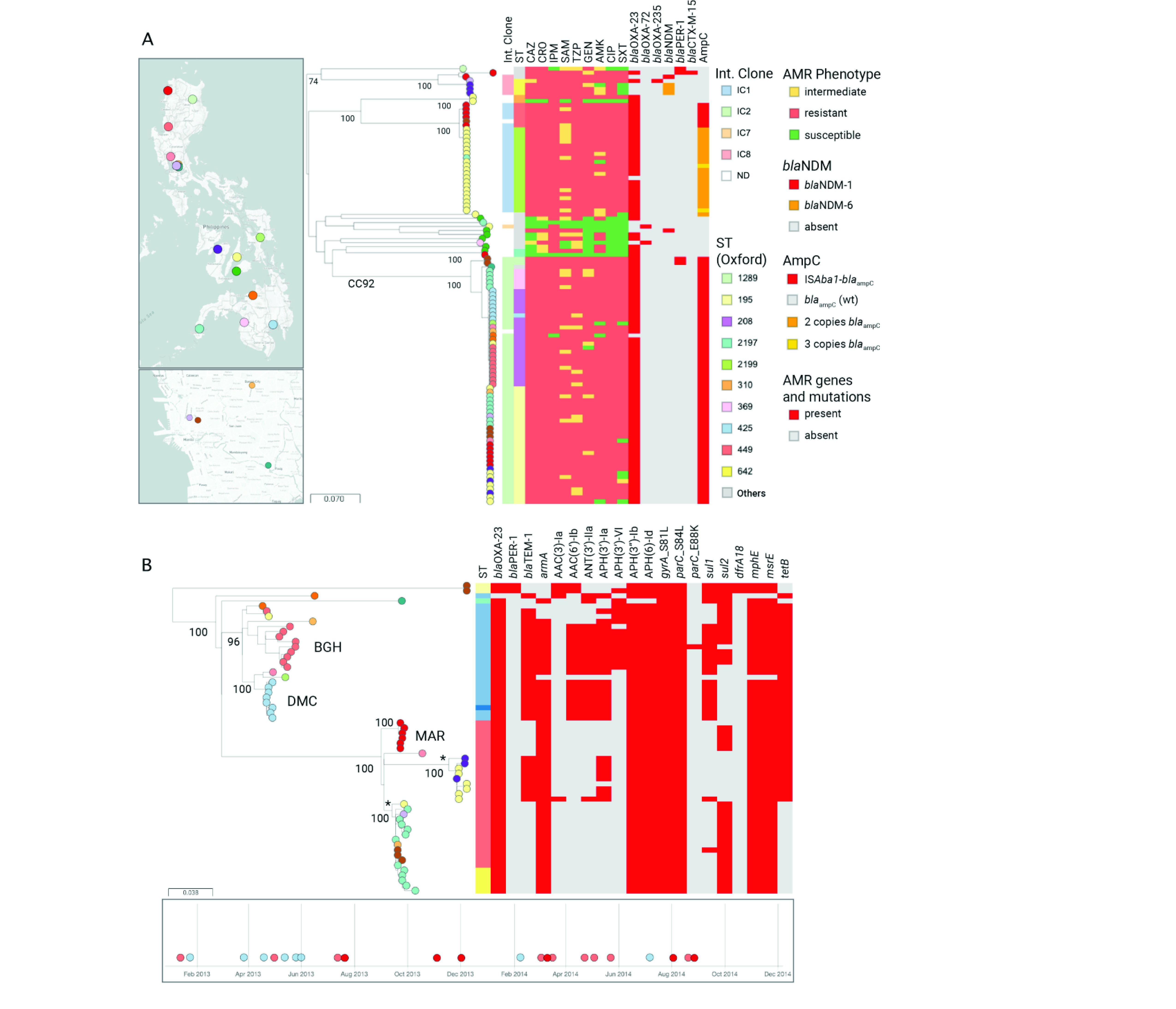
Genomic surveillance of A. baumannii from the Philippines, 2013–2014

The phylogenetic tree of 108 *A. baumannii* genomes showed that the population was composed of well defined clades that matched the distribution of the sequence types. The two main clonal groups were IC1 and IC2 (i.e. CC92; **Fig. 2a**), with a minor representation of IC8 and IC7. Isolates belonging to international clones were mostly XDR and are known to be responsible for disseminating AMR globally. The carbapenemase gene *bla***^OXA-23^** was found consistently in IC1 and IC2 genomes, and more sporadically in IC8 and nonclonal genomes. In contrast, the carbapenemase gene *bla***^NDM-6^** was found exclusively in three IC8 genomes from Corazon Locsin Montelibano Memorial Regional Hospital (MMH), while *bla***^NDM-1^** and *bla***^OXA-72^** were found only sporadically. Notably, isolates carrying IS*Aba1* inserted in the promoter of *bla***^ampC^** belonged to ST449 (IC1) or to CC92 (IC2), while isolates carrying two or three copies of the *bla***^ampC^** gene all belonged to a novel sequence type (now ST2199) found in the Vicente Sotto Memorial Medical Center (VSM) in the Visayas region and the Zamboanga City Medical Center in the Mindanao region (**Fig. 2a**).

[**insert** Figure 2]

The phylogenetic tree of 61 genomes from the prevalent XDR CC92 clone showed that most isolates were grouped into two clades represented by ST208 and single locus variant ST425 (bootstrap support, 96%) and by ST195 and single locus variant ST369 (bootstrap support, 100%) (**Fig. 2b**). Both ST208–ST425 and ST195–ST369 were found in hospitals from all three island groups (Luzon in the north, Visayas in the centre and Mindanao in the south), but their geographical distribution showed little overlap. The phylogeographical signal suggested there were both local outbreaks and interhospital dissemination (**Fig. 2b**). We investigated this further by counting the number of pairwise, nonrecombinant SNP differences between primary isolates from the same or different hospitals. First, we identified three intrahospital clusters (bootstrap support, 100%) of closely related isolates from BGH (ST208, 2–35 pairwise SNPs; *n* = 9), Southern Philippines Medical Center (DMC, ST208–ST425, 1–6 pairwise SNPs; *n* = 8) and Mariano Marcos Memorial Hospital and Medical Center (MAR, ST195, 0–3 pairwise SNPs; *n* = 6). The isolates within each of the three clusters carried identical or almost identical repertoires of resistance determinants, further supporting their clonal relationship. The isolation dates spanning more than 12 months suggested that these clonal lineages are possibly endemic to the hospitals, although regular introduction by colonized patients cannot be ruled out.

Next, we identified two clusters of closely related isolates from two or more hospitals. One cluster contained nine ST195 genomes from two hospitals in the Visayas region (MMH and VSM), with a median of only 5 pairwise SNP differences (range, 1–17) between isolates from different hospitals. The second one contained 18 ST195–ST369 genomes from six hospitals across three different regions, with a median of 25 pairwise SNP differences (range, 1–53). The clonal relationship between isolates from different hospitals within these two clusters is also supported by a similar complement of resistance determinants.

#### A. baumannii *from the Philippines in the global context*

**Figure 3:**
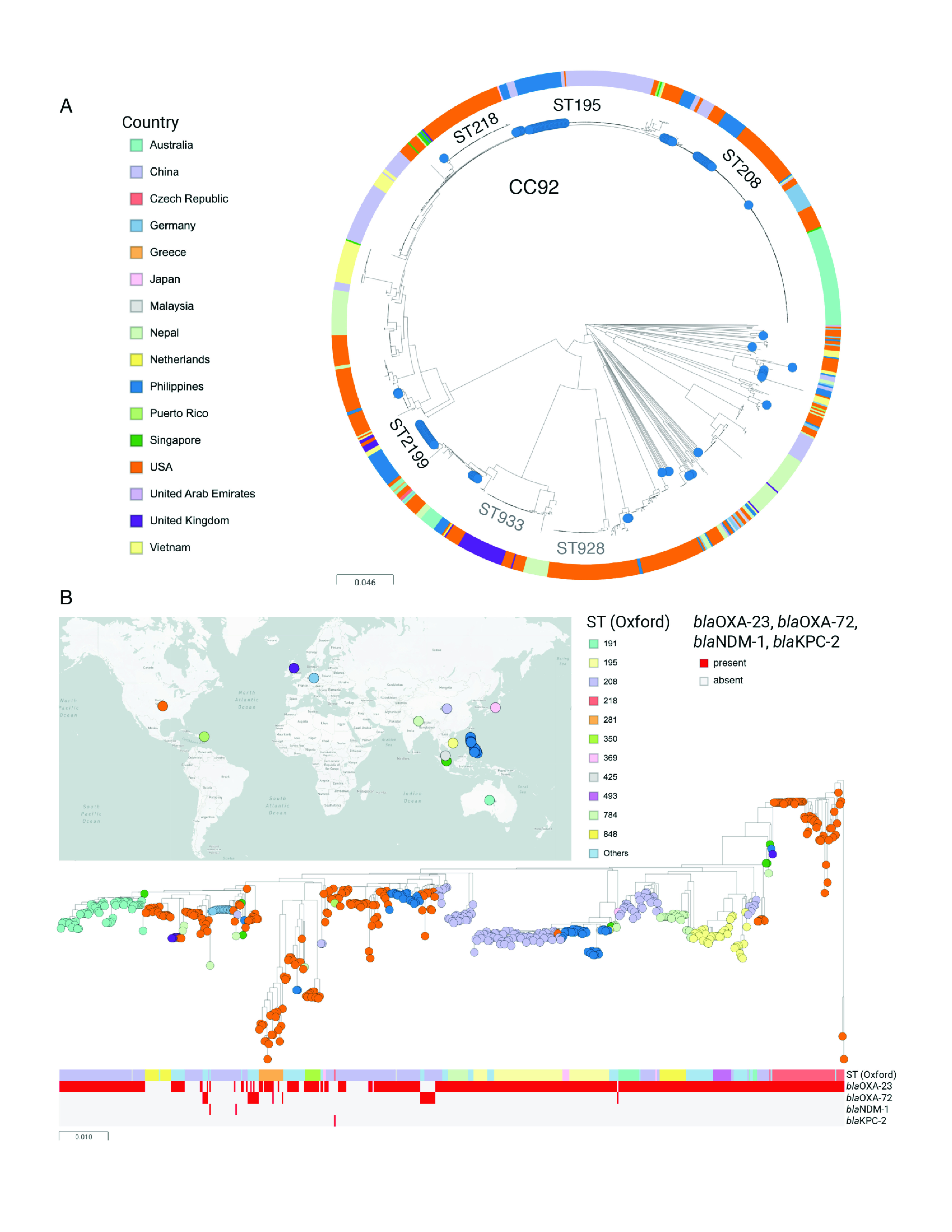
A. baumannii from the Philippines in global context

To place the retrospective collection of *A. baumannii* isolates from the Philippines in the context of the global population of this pathogen, we compared our genomes to 931 genomes publicly available from sequence data archives that have linked geographical and temporal information. The isolates were collected between 1982 and 2016, with 94.7% of the isolates collected from 2007 onwards. The public genomes belonged to 16 countries and were assigned to 154 sequence types. The population represented by the global genomes was substantially skewed towards genomes from the United States (40.5%) and belonging to CC92 (58.6%). The Philippine genomes were found in multiple branches of the tree, as expected by the diversity of sequence types, but they mostly formed discreet clusters within each branch without genomes from other countries interspersed (**Fig. 3a**). This suggests that the establishment of each clone in the Philippines is the result of one or only a few founding events.

[**insert** Figure 3]

To investigate in more detail the relationship to global genomes within CC92, a tree of 573 genomes was inferred from the alignment of nonrecombinant SNPs (**Fig. 3b**). The ST195–ST369 genomes from the Philippines clustered with genomes from China, Malaysia, Singapore, the United States and Viet Nam, while the ST208–ST425 genomes were related to genomes from China, Puerto Rico and the United States. However, the strong phylogeographical signal displayed by both the ST195–ST369 and the ST208–ST425 subtrees suggested a single founder event in the Philippines for each clone, followed by their expansion.

## Discussion

This study reports on the combined genomic and laboratory-based surveillance of *A. baumannii* in the Philippines during 2013–2014. The prevalence of carbapenem-resistant *A. baumannii* during this period was above 40%, and we therefore focused on characterizing these organisms. In *A. baumannii*, only low-level carbapenem resistance is mediated by the chromosomal OXA-51-like carbapenemase. The class D OXA-23 carbapenemase was the most prevalent acquired carbapenem resistance mechanism identified in this study, in line with global trends. (*24*) We also detected representatives from the OXA-235-like (*bla***^OXA-235^**) and the OXA-40-like (*bla***^OXA-72^**) groups, albeit in low frequency. No OXA-58-like carbapenemases were detected, as previously reported from other Asia–Pacific nations. (*25*) Importantly, we also detected the presence of the class B metallo-β-lactamases NDM-1 and NDM-6, which, unlike OXA-23, confer resistance to extended-spectrum cephalosporins as well as carbapenems. *A. baumannii* harbouring NDM-1 has been sporadically reported previously from other countries, (*26*-*28*) but NDM-6-carrying  *A. baumannii* has only recently been reported from Spain. (*29*) Resistance to extended-spectrum cephalosporins was mainly explained by the insertion of IS*Aba1* in the promoter of the intrinsic gene *bla***^ampC^**, which has been shown to lead to increased expression of the encoded cephalosporinase. (*30*) Identification of this mechanism represents an additional in silico query of the genomes, which is burdensome in the context of a public health reference laboratory, but omitting it would lead to high major error rates for genomic predictions of resistance to extended-spectrum cephalosporins.

Both IC1 and IC2, which are responsible for the spread of MDR and XDR phenotypes worldwide, (*24*, *31*) were found in the Philippines. However, IC2 was the predominant clonal type of *A. baumannii* in our study population, with ST195 and ST208 and their respective single locus variants found throughout the country. The global phylogenetic tree showed that these two lineages diverged before their establishment in the Philippines. The genetic relatedness of isolates from different hospitals and their similar complements of resistance determinants support the notion that their subsequent success was the result of clonal expansion and in-country geographical dissemination, rather than multiple introductions. This highlights the need for concerted infection prevention and control measures to contain the spread of high-risk clones. However, the limited number and disparate sampling of genomes from other countries in the region and the selective referral of carbapenem-resistant isolates to the reference laboratory by the sentinel sites limited our ability to capture the dynamics of these clones.

We also identified three ST195 and ST208 intrahospital clusters spanning more than 12 months  each. Resistance to antimicrobial drugs and to desiccation contribute to the survival of *A. baumannii* in the hospital environment, (*1*) and cross-contamination of hospital surfaces with MDR strains has been documented, particularly in the areas surrounding colonized or infected patients. (*32*, *33*) The ARSP does not currently include environmental samples, and thus it was not possible to connect the persistence of the intrahospital clusters to environmental contamination, which is a limitation of our study. Outbreaks of *A. baumannii* with *bla***^OXA-23^**, including of ST195 and ST208, have been reported from several countries, (*34*-*36*) and our study identified potential hospital outbreaks retrospectively. The resolution afforded by WGS was in stark contrast to the uniform resistance profiles of the isolates in our study, thus making cluster detection based on WGS rather than resistance profiles, of particular utility for carbapenem-resistant *A. baumannii*.

The assignment of isolates to an outbreak based on their genetic distance is key for effective patient containment and infection control during an ongoing investigation. Out of the three intrahospital IC2 clusters detected, the ST208 cluster from BGH displayed more genetic diversity than the other two, based on the number of pairwise SNP differences, opening the possibility that more than one closely related strain was circulating in the hospital. However, the absence of data on patient movement precluded the epidemiological investigation that would have aided in delineating the outbreaks, another limitation of our study. In addition, while the pairwise SNP differences are similar to those reported in other studies, (*35*, *37*-*39*) SNP thresholds are difficult to assess by comparison due to methodological differences, such as the use of core- versus whole-genome SNPs, the choice of reference genome for reference-based mapping of short reads, and the inclusion or exclusion of SNPs associated with recombination regions.

In conclusion, our retrospective genomic epidemiology study of carbapenem-resistant *A. baumannii* in the Philippines revealed that IC2 with OXA-23 is the main source of the increasing carbapenem resistance in the Philippines and that breaches in infection control and prevention likely contributed to its dissemination. WGS proved a useful tool for improving surveillance of  *A. baumannii*.
